# That Mystery—The Child

**Published:** 1897-07-17

**Authors:** 


### Modern Sociology.

### THAT MYSTERY—THE CHILD.

Fewer volumes on the bringing up of children appear now than were issued from the press a generation or two ago. For this children have much cause to be thankful, for though education may be a science—not one of the exact sciences, however—up-bringing in the widest sense is an art. Like other arts, the happy expert in it *nascitur non fit*, though cultivation may improve the native talent, and though, by cultivation and study, those even who have but little native bent may acquire some skill in the art, as an ear for music may be gained and improved. The conception of this is growing in the public mind, and thereby has killed those old-fashioned manuals which laid down rules for the turning out of intelligent and virtuous men and women as the cookery books give rules for the turning out of plum puddings.

There are, indeed, certain qualities which we would all like our children to possess—honesty, courage, truthfulness, discretion, and so forth; but there is no one method by which these qualities may be implanted in all breasts. Besides, so great a gulf lies between the ideas of the adult and those of the child that advice given in all good faith may have the most surprising results. You advise your timid boy to be brave, and, with the intention of obeying you, he so overcomes his natural terrors as to climb the highest tree in the neighbourhood or venture out in a leaky boat; or you bid him be truthful, and he brings you stories of school-room and nursery, with overheard bits of kitchen gossip, till you think he is growing into a sneak and a tell-tale. Then you impress on him the duty of loyal silence, of reticence concerning other people's affairs, with the result that he lies through thick and thin, bears undeserved accusations and even undeserved beatings, to save a friend or even a servant from the consequences of their misdeeds. For the child is an extremist in all things. He knows black and white, but his moral colour sense takes no cognizance of grey—of those limitations and compromises by which we guard our virtues and seek to save ourselves from "the defects of our qualities." It is not strange if, bewildered at being rebuked for everything he does, he gives up trying to be "good," and is merely natural, which, indeed, may produce quite as satisfactory results.

One thing which the old hand-books ignored was the difference between the temperaments of different children. "I brought them all up in exactly the same way," says a weeping mother when one of her children turns out a sinner while another is a saint. But they were not the same in nature, and therefore the same treatment produced different results. It is not necessary to lay drains in the Sahara, though it is most desirable to do so in the fens.

What is necessary is to study the child himself, his virtues and his vices, remembering, however, that the latter are generally unconscious, and that before punishing them as moral defects you must instruct the child in the fact that they are such. Thus, most children are liars and thieves by nature. The theft is easily explained; they have no notion of property in the social sense. They are born individualists. They see a thing, they desire it, they take it; and it has to be explained to them—by words, not by blows—that the producer has certain rights in his product, and that if each took exactly what pleased him without reference to the rights and feelings of others, society, even the society of the nursery, would be reduced to a state of warfare, and he—the child—being the weakest might come off the worst in the fray. Thus early may the moral of economics be explained.

Lying is a more complicated phenomenon. Sometimes it is purely imaginative: "I was a little bird," or "I was a fairy, and lived in a bluebell." But often enough it takes a more serious aspect: "I didn't break the cup," the fragments of which lie on the floor; or "I didn't take the jam," with which mouth and hands are stained. Now, instead of reverting to memories of Eve and original sin, it is well to realise that these falsehoods are mere examples of the instinct of self-preservation. The child has learned in his brief experience of life that the breaking of cups and the unpermitted devouring of jam result in punishment, and instinctively he shelters himself behind a denial of responsibility. Older people do the same, though perhaps less clumsily. Therefore, distressed mother, instead of whipping your child for lying, explain to him the meanness and injustice of letting other persons suffer for an act, admittedly blameworthy, of which they were innocent. Very few children are wilfully unjust. They wish to shelter themselves; but in almost all cases they will own their guilt rather than see others suffer.

Stupidity, real and apparent, in children presents a difficult study. There comes a time when the colt must be put in the harness, the child begin to study. As neither task is natural to the animal involved, it is almost impossible to accomplish it without a certain severity. The thing to be desired in both cases is that the severity may be no more than sufficient, that the powers of each creature may be guided in the right direction without being cramped and maimed. And therefore the application of whip, or bit, or spur must be accompanied by careful study of the animal. You can never make a cart-horse win the Derby, and you can never make a stupid child a clever one; but you can find out wherein his stupidity lies, and what compensation Nature has afforded him. It is only in comparatively recent years that we have begun to perceive how much tone-deafness, colour-blindness, or myopia may have to do with an apparent dulness which was too often set down as the result of inattention. Even where no such easily-diagnosed defect exists one must admit such differences as puzzle the wisest. Against the phenomenal "calculating boy," to whom all arithmetical problems are as nothing, you put the child who can scarcely grasp the fact that two and two make four as an abstract idea. Yet he may be no more stupid than the other, but only of a more materialisti temper, which realises things only when set in visible shape before it. The natural tendency of schoolmasters is to condemn as stupid the child who is dull in things scholastic. Life often reverses the schoolmaster's verdict, and shows that the so-called dulness was intelligence which had not yet found its proper channel.

